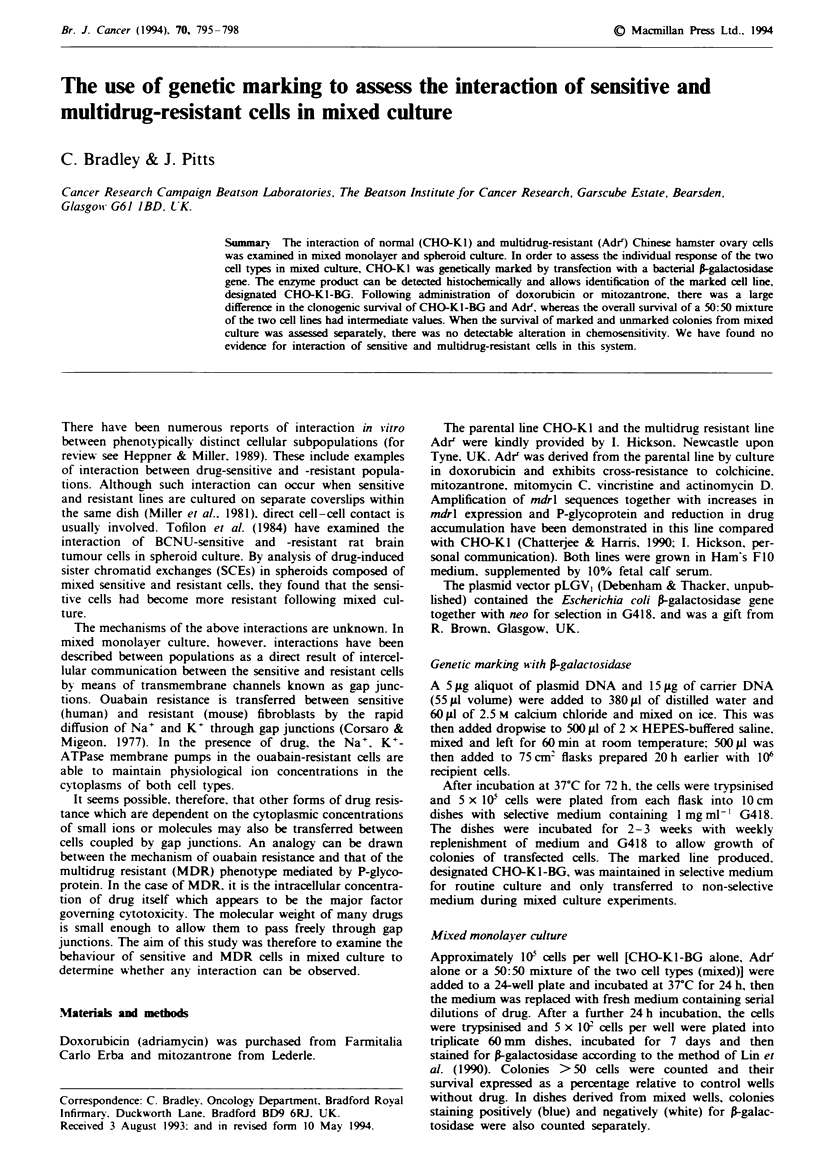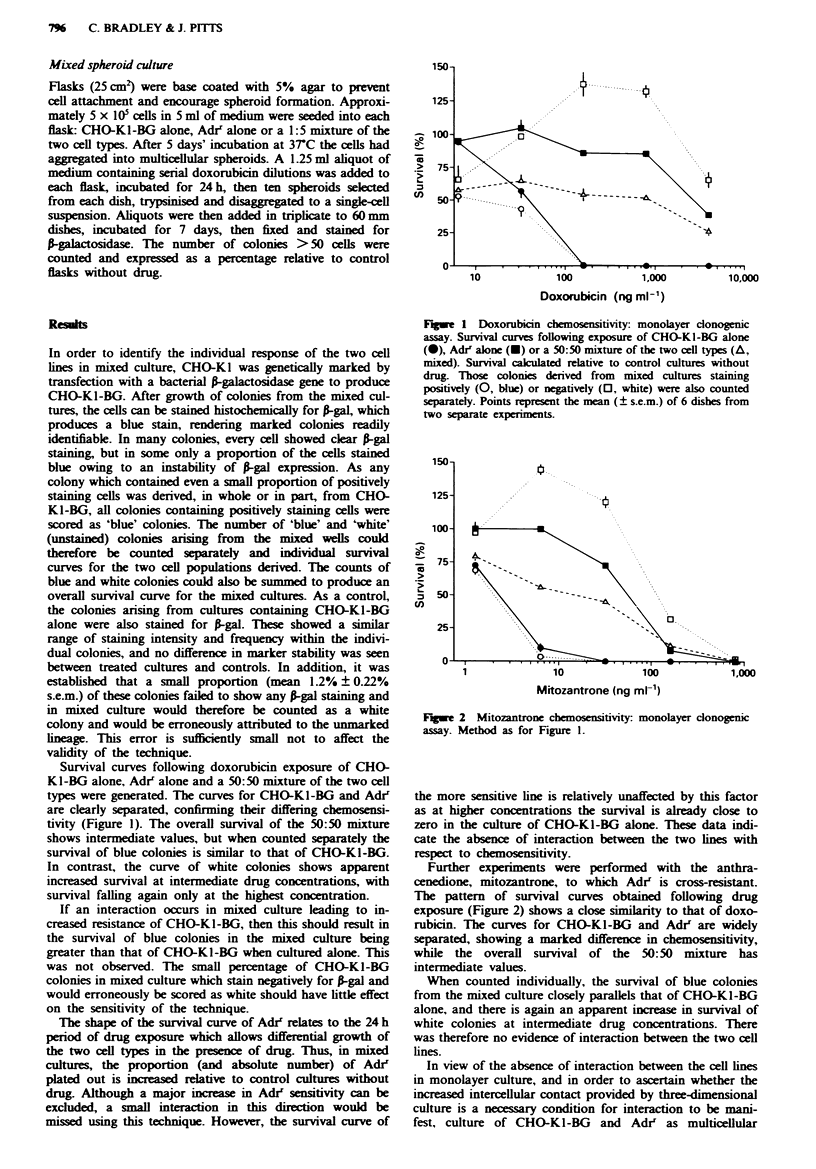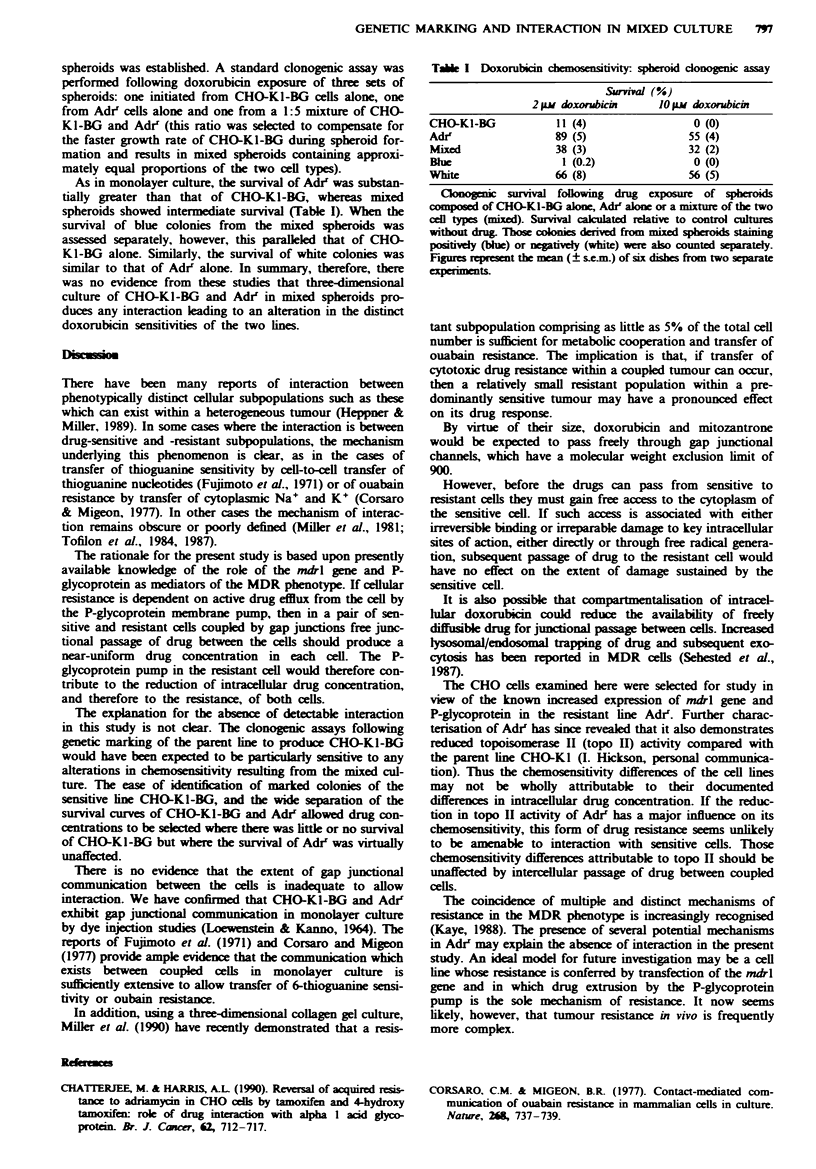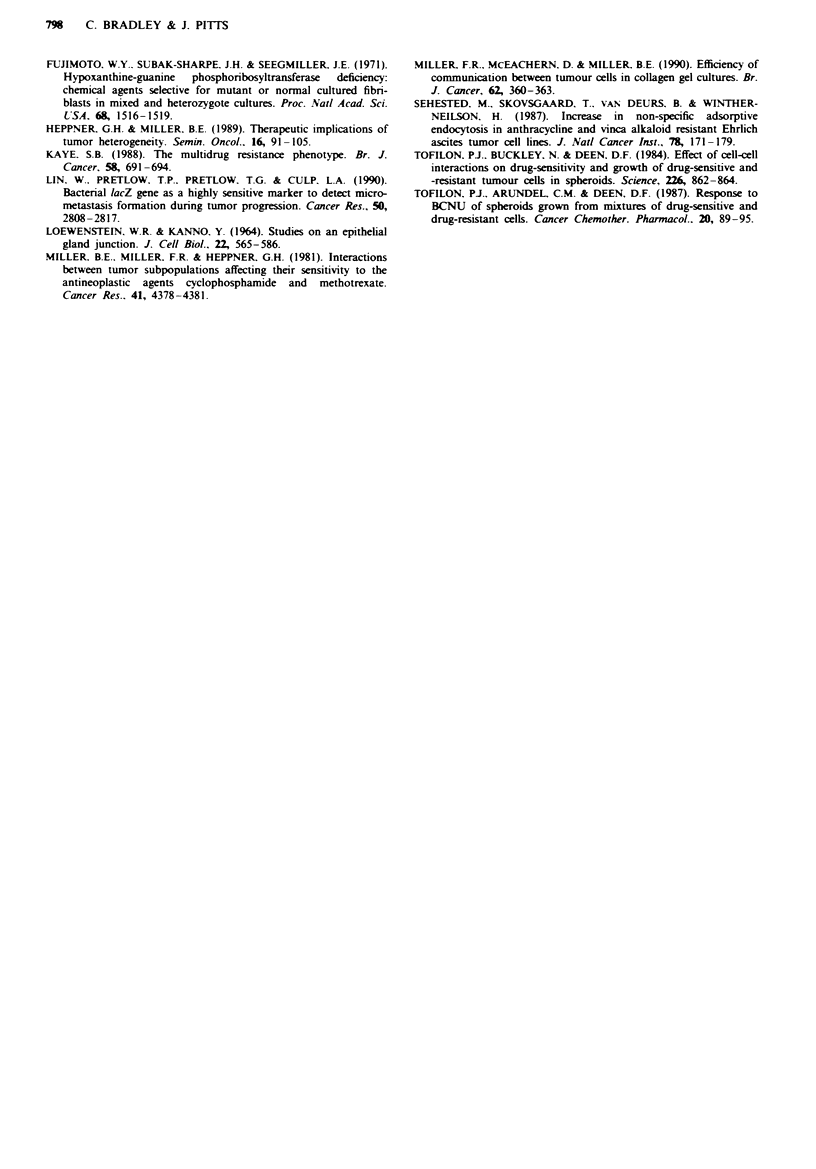# The use of genetic marking to assess the interaction of sensitive and multidrug-resistant cells in mixed culture.

**DOI:** 10.1038/bjc.1994.401

**Published:** 1994-11

**Authors:** C. Bradley, J. Pitts

**Affiliations:** Cancer Research Campaign Beatson Laboratories, Beatson Institute for Cancer Research, Bearsden, Glasgow, UK.

## Abstract

The interaction of normal (CHO-K1) and multidrug-resistant (Adrr) Chinese hamster ovary cells was examined in mixed monolayer and spheroid culture. In order to assess the individual response of the two cell types in mixed culture, CHO-K1 was genetically marked by transfection with a bacterial beta-galactosidase gene. The enzyme product can be detected histochemically and allows identification of the marked cell line, designated CHO-K1-BG. Following administration of doxorubicin or mitozantrone, there was a large difference in the clonogenic survival of CHO-K1-BG and Adrr, whereas the overall survival of a 50:50 mixture of the two cell lines had intermediate values. When the survival of marked and unmarked colonies from mixed culture was assessed separately, there was no detectable alteration in chemosensitivity. We have found no evidence for interaction of sensitive and multidrug-resistant cells in this system.


Br. J. Cancer (1994), 70, 795-798 © Macmillan Press Ltd., 1994

### The use of genetic marking to assess the interaction of sensitive and multidrug-resistant cells in mixed culture

C. Bradley & J. Pitts

*Cancer Research Campaign Beatson Laboratories, The Beatson Institute for Cancer Research, Garscube Estate, Bearsden, Glasgow G61 1BD, UK.*

**Summary** The interaction of normal (CHO-K1) and multidrug-resistant ($Adr^r$) Chinese hamster ovary cells was examined in mixed monolayer and spheroid culture. In order to assess the individual response of the two cell types in mixed culture, CHO-K1 was genetically marked by transfection with a bacterial β-galactosidase gene. The enzyme product can be detected histochemically and allows identification of the marked cell line, designated CHO-K1-BG. Following administration of doxorubicin or mitozantrone, there was a large difference in the clonogenic survival of CHO-K1-BG and $Adr^r$, whereas the overall survival of a 50:50 mixture of the two cell lines had intermediate values. When the survival of marked and unmarked colonies from mixed culture was assessed separately, there was no detectable alteration in chemosensitivity. We have found no evidence for interaction of sensitive and multidrug-resistant cells in this system.

There have been numerous reports of interaction *in vitro* between phenotypically distinct cellular subpopulations (for review see Heppner & Miller, 1989). These include examples of interaction between drug-sensitive and -resistant populations. Although such interaction can occur when sensitive and resistant lines are cultured on separate coverslips within the same dish (Miller *et al.*, 1981), direct cell-cell contact is usually involved. Tofilon *et al.* (1984) have examined the interaction of BCNU-sensitive and -resistant rat brain tumour cells in spheroid culture. By analysis of drug-induced sister chromatid exchanges (SCEs) in spheroids composed of mixed sensitive and resistant cells, they found that the sensitive cells had become more resistant following mixed culture.

The mechanisms of the above interactions are unknown. In mixed monolayer culture, however, interactions have been described between populations as a direct result of intercellular communication between the sensitive and resistant cells by means of transmembrane channels known as gap junctions. Ouabain resistance is transferred between sensitive (human) and resistant (mouse) fibroblasts by the rapid diffusion of $Na^+$ and $K^+$ through gap junctions (Corsaro & Migeon, 1977). In the presence of drug, the $Na^+$, $K^+$-ATPase membrane pumps in the ouabain-resistant cells are able to maintain physiological ion concentrations in the cytoplasms of both cell types.

It seems possible, therefore, that other forms of drug resistance which are dependent on the cytoplasmic concentrations of small ions or molecules may also be transferred between cells coupled by gap junctions. An analogy can be drawn between the mechanism of ouabain resistance and that of the multidrug resistant (MDR) phenotype mediated by P-glycoprotein. In the case of MDR, it is the intracellular concentration of drug itself which appears to be the major factor governing cytotoxicity. The molecular weight of many drugs is small enough to allow them to pass freely through gap junctions. The aim of this study was therefore to examine the behaviour of sensitive and MDR cells in mixed culture to determine whether any interaction can be observed.

### Materials and methods

Doxorubicin (adriamycin) was purchased from Farmitalia Carlo Erba and mitozantrone from Lederle.

Correspondence: C. Bradley, Oncology Department, Bradford Royal Infirmary, Duckworth Lane, Bradford BD9 6RJ, UK.
Received 3 August 1993; and in revised form 10 May 1994.

The parental line CHO-K1 and the multidrug resistant line $Adr^r$ were kindly provided by I. Hickson, Newcastle upon Tyne, UK. $Adr^r$ was derived from the parental line by culture in doxorubicin and exhibits cross-resistance to colchicine, mitozantrone, mitomycin C, vincristine and actinomycin D. Amplification of *mdr*1 sequences together with increases in *mdr*1 expression and P-glycoprotein and reduction in drug accumulation have been demonstrated in this line compared with CHO-K1 (Chatterjee & Harris, 1990; I. Hickson, personal communication). Both lines were grown in Ham's F10 medium, supplemented by 10% fetal calf serum.

The plasmid vector $pLGV_1$ (Debenham & Thacker, unpublished) contained the *Escherichia coli* β-galactosidase gene together with *neo* for selection in G418, and was a gift from R. Brown, Glasgow, UK.

### *Genetic marking with β-galactosidase*

A 5 μg aliquot of plasmid DNA and 15 μg of carrier DNA (55 μl volume) were added to 380 μl of distilled water and 60 μl of 2.5 M calcium chloride and mixed on ice. This was then added dropwise to 500 μl of 2 × HEPES-buffered saline, mixed and left for 60 min at room temperature; 500 μl was then added to 75 $cm^2$ flasks prepared 20 h earlier with $10^6$ recipient cells.

After incubation at 37°C for 72 h, the cells were trypsinised and $5 \times 10^5$ cells were plated from each flask into 10 cm dishes with selective medium containing 1 mg $ml^{-1}$ G418. The dishes were incubated for 2-3 weeks with weekly replenishment of medium and G418 to allow growth of colonies of transfected cells. The marked line produced, designated CHO-K1-BG, was maintained in selective medium for routine culture and only transferred to non-selective medium during mixed culture experiments.

### *Mixed monolayer culture*

Approximately $10^5$ cells per well [CHO-K1-BG alone, $Adr^r$ alone or a 50:50 mixture of the two cell types (mixed)] were added to a 24-well plate and incubated at 37°C for 24 h, then the medium was replaced with fresh medium containing serial dilutions of drug. After a further 24 h incubation, the cells were trypsinised and $5 \times 10^2$ cells per well were plated into triplicate 60 mm dishes, incubated for 7 days and then stained for β-galactosidase according to the method of Lin *et al.* (1990). Colonies >50 cells were counted and their survival expressed as a percentage relative to control wells without drug. In dishes derived from mixed wells, colonies staining positively (blue) and negatively (white) for β-galactosidase were also counted separately.

*Mixed spheroid culture*

Flasks (25 $cm^2$) were base coated with 5% agar to prevent cell attachment and encourage spheroid formation. Approximately $5 \times 10^5$ cells in 5 ml of medium were seeded into each flask: CHO-K1-BG alone, $Adr^r$ alone or a 1:5 mixture of the two cell types. After 5 days' incubation at 37°C the cells had aggregated into multicellular spheroids. A 1.25 ml aliquot of medium containing serial doxorubicin dilutions was added to each flask, incubated for 24 h, then ten spheroids selected from each dish, trypsinised and disaggregated to a single-cell suspension. Aliquots were then added in triplicate to 60 mm dishes, incubated for 7 days, then fixed and stained for β-galactosidase. The number of colonies >50 cells were counted and expressed as a percentage relative to control flasks without drug.

### Results

In order to identify the individual response of the two cell lines in mixed culture, CHO-K1 was genetically marked by transfection with a bacterial β-galactosidase gene to produce CHO-K1-BG. After growth of colonies from the mixed cultures, the cells can be stained histochemically for β-gal, which produces a blue stain, rendering marked colonies readily identifiable. In many colonies, every cell showed clear β-gal staining, but in some only a proportion of the cells stained blue owing to an instability of β-gal expression. As any colony which contained even a small proportion of positively staining cells was derived, in whole or in part, from CHO-K1-BG, all colonies containing positively staining cells were scored as 'blue' colonies. The number of 'blue' and 'white' (unstained) colonies arising from the mixed wells could therefore be counted separately and individual survival curves for the two cell populations derived. The counts of blue and white colonies could also be summed to produce an overall survival curve for the mixed cultures. As a control, the colonies arising from cultures containing CHO-K1-BG alone were also stained for β-gal. These showed a similar range of staining intensity and frequency within the individual colonies, and no difference in marker stability was seen between treated cultures and controls. In addition, it was established that a small proportion (mean 1.2% ± 0.22% s.e.m.) of these colonies failed to show any β-gal staining and in mixed culture would therefore be counted as a white colony and would be erroneously attributed to the unmarked lineage. This error is sufficiently small not to affect the validity of the technique.

Survival curves following doxorubicin exposure of CHO-K1-BG alone, $Adr^r$ alone and a 50:50 mixture of the two cell types were generated. The curves for CHO-K1-BG and $Adr^r$ are clearly separated, confirming their differing chemosensitivity (Figure 1). The overall survival of the 50:50 mixture shows intermediate values, but when counted separately the survival of blue colonies is similar to that of CHO-K1-BG. In contrast, the curve of white colonies shows apparent increased survival at intermediate drug concentrations, with survival falling again only at the highest concentration.

If an interaction occurs in mixed culture leading to increased resistance of CHO-K1-BG, then this should result in the survival of blue colonies in the mixed culture being greater than that of CHO-K1-BG when cultured alone. This was not observed. The small percentage of CHO-K1-BG colonies in mixed culture which stain negatively for β-gal and would erroneously be scored as white should have little effect on the sensitivity of the technique.

The shape of the survival curve of $Adr^r$ relates to the 24 h period of drug exposure which allows differential growth of the two cell types in the presence of drug. Thus, in mixed cultures, the proportion (and absolute number) of $Adr^r$ plated out is increased relative to control cultures without drug. Although a major increase in $Adr^r$ sensitivity can be excluded, a small interaction in this direction would be missed using this technique. However, the survival curve of the more sensitive line is relatively unaffected by this factor as at higher concentrations the survival is already close to zero in the culture of CHO-K1-BG alone. These data indicate the absence of interaction between the two lines with respect to chemosensitivity.

**Figure 1** Doxorubicin chemosensitivity: monolayer clonogenic assay. Survival curves following exposure of CHO-K1-BG alone (●), $Adr^r$ alone (■) or a 50:50 mixture of the two cell types (Δ, mixed). Survival calculated relative to control cultures without drug. Those colonies derived from mixed cultures staining positively (O, blue) or negatively (□, white) were also counted separately. Points represent the mean (± s.e.m.) of 6 dishes from two separate experiments.

**Figure 2** Mitozantrone chemosensitivity: monolayer clonogenic assay. Method as for Figure 1.

Further experiments were performed with the anthracenedione, mitozantrone, to which $Adr^r$ is cross-resistant. The pattern of survival curves obtained following drug exposure (Figure 2) shows a close similarity to that of doxorubicin. The curves for CHO-K1-BG and $Adr^r$ are widely separated, showing a marked difference in chemosensitivity, while the overall survival of the 50:50 mixture has intermediate values.

When counted individually, the survival of blue colonies from the mixed culture closely parallels that of CHO-K1-BG alone, and there is again an apparent increase in survival of white colonies at intermediate drug concentrations. There was therefore no evidence of interaction between the two cell lines.

In view of the absence of interaction between the cell lines in monolayer culture, and in order to ascertain whether the increased intercellular contact provided by three-dimensional culture is a necessary condition for interaction to be manifest, culture of CHO-K1-BG and $Adr^r$ as multicellular spheroids was established. A standard clonogenic assay was performed following doxorubicin exposure of three sets of spheroids: one initiated from CHO-K1-BG cells alone, one from Adr$^r$ cells alone and one from a 1:5 mixture of CHO-K1-BG and Adr$^r$ (this ratio was selected to compensate for the faster growth rate of CHO-K1-BG during spheroid formation and results in mixed spheroids containing approximately equal proportions of the two cell types).

As in monolayer culture, the survival of Adr$^r$ was substantially greater than that of CHO-K1-BG, whereas mixed spheroids showed intermediate survival (Table I). When the survival of blue colonies from the mixed spheroids was assessed separately, however, this paralleled that of CHO-K1-BG alone. Similarly, the survival of white colonies was similar to that of Adr$^r$ alone. In summary, therefore, there was no evidence from these studies that three-dimensional culture of CHO-K1-BG and Adr$^r$ in mixed spheroids produces any interaction leading to an alteration in the distinct doxorubicin sensitivities of the two lines.

**Table I** Doxorubicin chemosensitivity: spheroid clonogenic assay

| | *Survival (%)* | |
|---|---|---|
| | *2 μM doxorubicin* | *10 μM doxorubicin* |
| CHO-K1-BG | 11 (4) | 0 (0) |
| Adr$^r$ | 89 (5) | 55 (4) |
| Mixed | 38 (3) | 32 (2) |
| Blue | 1 (0.2) | 0 (0) |
| White | 66 (8) | 56 (5) |

Clonogenic survival following drug exposure of spheroids composed of CHO-K1-BG alone, Adr$^r$ alone or a mixture of the two cell types (mixed). Survival calculated relative to control cultures without drug. Those colonies derived from mixed spheroids staining positively (blue) or negatively (white) were also counted separately. Figures represent the mean (± s.e.m.) of six dishes from two separate experiments.

### Discussion

There have been many reports of interaction between phenotypically distinct cellular subpopulations such as these which can exist within a heterogeneous tumour (Heppner & Miller, 1989). In some cases where the interaction is between drug-sensitive and -resistant subpopulations, the mechanism underlying this phenomenon is clear, as in the cases of transfer of thioguanine sensitivity by cell-to-cell transfer of thioguanine nucleotides (Fujimoto *et al.*, 1971) or of ouabain resistance by transfer of cytoplasmic $Na^+$ and $K^+$ (Corsaro & Migeon, 1977). In other cases the mechanism of interaction remains obscure or poorly defined (Miller *et al.*, 1981; Tofilon *et al.*, 1984, 1987).

The rationale for the present study is based upon presently available knowledge of the role of the *mdr*1 gene and P-glycoprotein as mediators of the MDR phenotype. If cellular resistance is dependent on active drug efflux from the cell by the P-glycoprotein membrane pump, then in a pair of sensitive and resistant cells coupled by gap junctions free junctional passage of drug between the cells should produce a near-uniform drug concentration in each cell. The P-glycoprotein pump in the resistant cell would therefore contribute to the reduction of intracellular drug concentration, and therefore to the resistance, of both cells.

The explanation for the absence of detectable interaction in this study is not clear. The clonogenic assays following genetic marking of the parent line to produce CHO-K1-BG would have been expected to be particularly sensitive to any alterations in chemosensitivity resulting from the mixed culture. The ease of identification of marked colonies of the sensitive line CHO-K1-BG, and the wide separation of the survival curves of CHO-K1-BG and Adr$^r$ allowed drug concentrations to be selected where there was little or no survival of CHO-K1-BG but where the survival of Adr$^r$ was virtually unaffected.

There is no evidence that the extent of gap junctional communication between the cells is inadequate to allow interaction. We have confirmed that CHO-K1-BG and Adr$^r$ exhibit gap junctional communication in monolayer culture by dye injection studies (Loewenstein & Kanno, 1964). The reports of Fujimoto *et al.* (1971) and Corsaro and Migeon (1977) provide ample evidence that the communication which exists between coupled cells in monolayer culture is sufficiently extensive to allow transfer of 6-thioguanine sensitivity or oubain resistance.

In addition, using a three-dimensional collagen gel culture, Miller *et al.* (1990) have recently demonstrated that a resistant subpopulation comprising as little as 5% of the total cell number is sufficient for metabolic cooperation and transfer of ouabain resistance. The implication is that, if transfer of cytotoxic drug resistance within a coupled tumour can occur, then a relatively small resistant population within a predominantly sensitive tumour may have a pronounced effect on its drug response.

By virtue of their size, doxorubicin and mitozantrone would be expected to pass freely through gap junctional channels, which have a molecular weight exclusion limit of 900.

However, before the drugs can pass from sensitive to resistant cells they must gain free access to the cytoplasm of the sensitive cell. If such access is associated with either irreversible binding or irreparable damage to key intracellular sites of action, either directly or through free radical generation, subsequent passage of drug to the resistant cell would have no effect on the extent of damage sustained by the sensitive cell.

It is also possible that compartmentalisation of intracellular doxorubicin could reduce the availability of freely diffusible drug for junctional passage between cells. Increased lysosomal/endosomal trapping of drug and subsequent exocytosis has been reported in MDR cells (Sehested *et al.*, 1987).

The CHO cells examined here were selected for study in view of the known increased expression of *mdr*1 gene and P-glycoprotein in the resistant line Adr$^r$. Further characterisation of Adr$^r$ has since revealed that it also demonstrates reduced topoisomerase II (topo II) activity compared with the parent line CHO-K1 (I. Hickson, personal communication). Thus the chemosensitivity differences of the cell lines may not be wholly attributable to their documented differences in intracellular drug concentration. If the reduction in topo II activity of Adr$^r$ has a major influence on its chemosensitivity, this form of drug resistance seems unlikely to be amenable to interaction with sensitive cells. Those chemosensitivity differences attributable to topo II should be unaffected by intercellular passage of drug between coupled cells.

The coincidence of multiple and distinct mechanisms of resistance in the MDR phenotype is increasingly recognised (Kaye, 1988). The presence of several potential mechanisms in Adr$^r$ may explain the absence of interaction in the present study. An ideal model for future investigation may be a cell line whose resistance is conferred by transfection of the *mdr*1 gene and in which drug extrusion by the P-glycoprotein pump is the sole mechanism of resistance. It now seems likely, however, that tumour resistance *in vivo* is frequently more complex.

### References

CHATTERJEE, M. & HARRIS, A.L. (1990). Reversal of acquired resistance to adriamycin in CHO cells by tamoxifen and 4-hydroxy tamoxifen: role of drug interaction with alpha 1 acid glycoprotein. *Br. J. Cancer*, **62**, 712–717.

CORSARO, C.M. & MIGEON, B.R. (1977). Contact-mediated communication of ouabain resistance in mammalian cells in culture. *Nature*, **268**, 737–739.

FUJIMOTO, W.Y., SUBAK-SHARPE, J.H. & SEEGMILLER, J.E. (1971). Hypoxanthine-guanine phosphoribosyltransferase deficiency: chemical agents selective for mutant or normal cultured fibriblasts in mixed and heterozygote cultures. *Proc. Natl Acad. Sci. USA*, **68**, 1516-1519.

HEPPNER, G.H. & MILLER, B.E. (1989). Therapeutic implications of tumor heterogeneity. *Semin. Oncol.*, **16**, 91-105.

KAYE, S.B. (1988). The multidrug resistance phenotype. *Br. J. Cancer*, **58**, 691-694.

LIN, W., PRETLOW, T.P., PRETLOW, T.G. & CULP, L.A. (1990). Bacterial *lacZ* gene as a highly sensitive marker to detect micrometastasis formation during tumor progression. *Cancer Res.*, **50**, 2808-2817.

LOEWENSTEIN, W.R. & KANNO, Y. (1964). Studies on an epithelial gland junction. *J. Cell Biol.*, **22**, 565-586.

MILLER, B.E., MILLER, F.R. & HEPPNER, G.H. (1981). Interactions between tumor subpopulations affecting their sensitivity to the antineoplastic agents cyclophosphamide and methotrexate. *Cancer Res.*, **41**, 4378-4381.

MILLER, F.R., MCEACHERN, D. & MILLER, B.E. (1990). Efficiency of communication between tumour cells in collagen gel cultures. *Br. J. Cancer*, **62**, 360-363.

SEHESTED, M., SKOVSGAARD, T., VAN DEURS, B. & WINTHER-NEILSON, H. (1987). Increase in non-specific adsorptive endocytosis in anthracycline and vinca alkaloid resistant Ehrlich ascites tumor cell lines. *J. Natl Cancer Inst.*, **78**, 171-179.

TOFILON, P.J., BUCKLEY, N. & DEEN, D.F. (1984). Effect of cell-cell interactions on drug-sensitivity and growth of drug-sensitive and -resistant tumour cells in spheroids. *Science*, **226**, 862-864.

TOFILON, P.J., ARUNDEL, C.M. & DEEN, D.F. (1987). Response to BCNU of spheroids grown from mixtures of drug-sensitive and drug-resistant cells. *Cancer Chemother. Pharmacol.*, **20**, 89-95.